# Is there a gender-dependent negative inotropic effect of Methylene blue on the contractile performance on the level of myofilaments?

**DOI:** 10.1186/1749-8090-10-S1-A258

**Published:** 2015-12-16

**Authors:** Constanze Bening, Helge Weiler, Nicole Stumpf, Christian-Friedrich Vahl

**Affiliations:** 1 Department of Cardiothoracic -and Vascular Surgery, University Hospital Mainz, 55131, Mainz, Germany; 2 Department of Cardiology, University Hospital Frankfurt, 60596, Frankfurt, Germany

## Background/Introduction

Methylene blue (MB) is a frequently used in patients with systemic inflammatory response syndrome with the effect of an increase in systemic vascular resistance. Recent studies assume that MB improves contractility and sensitizes myocardium to catecholamines.

## Aims/Objectives

But there are no data on human cardiac tissue showing whether MB has any impact on cardiac performance on the level of contractile apparatus and whether there is any gender difference.

## Method

Right auricle tissue from 12 patients (6 male and 6 female patients) undergoing elective cardiac surgery (aortocoronary bypass grafting) was obtained prior to right atrial cannulation. The tissue was prepared in 24 hours lasting procedure, washing out all membrane-dependent properties and enzymes. The fibers were dissected into small bundles and exposed to a gradual increase of calcium concentration starting with a pCa 6.0 up to pCa 4.5. The fibers first underwent an increase of calcium concentration until pCa 4.5. Afterwards the fibers are relaxed in calcium-free solution until steady state is achieved. Afterwards a second cycle is started with pCa 6.0 and 107 mol MB is added to every step of increasing calcium concentration. We performed 3 experiments with different fibers in each cycle and each patient (n = 30 in each patient with and without MB).

## Results

1. Skinned human fibers exposed to MB develop less force than those without MB 2.2 ± 0.02 mN versus 2.6 ± 0.1 mN. 2. Male fibers develop significant less force in presence of MB compared to those without (2.1 ± 0.2 mN versus 2.5 ± 0.7 mN, p 0.0002) 3. In presence of MB there was an impact of gender: male fibers: 2.1 ± 0.1 mN versus 2.7 ± 0.7 mN, p 0.02, This gender effect was not given in skinned fibers without exposure to MB.

## Discussion/Conclusion

Our data showed that MB has a negative inotropic effect on contractile performance of skinned human atrial fibers. We observed reduced absolute forces after exposure to MB and additionally there is some evidence for a gender-dependent effect on the contractile apparatus in presence of MB.

